# Nitric Oxide Signaling in Plants

**DOI:** 10.3390/plants9111550

**Published:** 2020-11-12

**Authors:** John T. Hancock

**Affiliations:** Department of Applied Sciences, University of the West of England, Bristol BS16 1QY, UK; john.hancock@uwe.ac.uk; Tel.: +44-(0)117-328-2475

**Keywords:** nitrate reductase, nitration, nitric oxide, reactive oxygen species, stress responses, *S*-nitrosation, *S*-nitrosylation, SNO-reductase, thiol modification

## Abstract

Nitric oxide (NO) is an integral part of cell signaling mechanisms in animals and plants. In plants, its enzymatic generation is still controversial. Evidence points to nitrate reductase being important, but the presence of a nitric oxide synthase-like enzyme is still contested. Regardless, NO has been shown to mediate many developmental stages in plants, and to be involved in a range of physiological responses, from stress management to stomatal aperture closure. Downstream from its generation are alterations of the actions of many cell signaling components, with post-translational modifications of proteins often being key. Here, a collection of papers embraces the differing aspects of NO metabolism in plants.

## 1. Introduction

Nitric oxide (NO) is now well acknowledged as an instrumental signaling molecule in both plants and animals [1]. First recognized as important as a signal in the control of vascular tone [2], its role in plants came to prominence in the late 1990s [3,4,5]. The forty years of research into NO in plants has just been highlighted by a review by Kolbert et al. [6].

In plants, NO has been found to be involved in a wide range of developmental stages and physiological responses. For example, NO has been found to be generated during pollination and pollen tube growth [7,8,9], seed germination [10], root development [11,12], and stomatal aperture control [13,14]. It is also instrumental in the orchestration of responses to stress in plants [15], including to heavy metals such as cadmium [16], salt [17], temperature [18], light [19] and pathogens [20].

NO in animals is known to be generated by several sources, but primarily nitric oxide synthase (NOS) is the enzyme which has a dominant role in NO accumulation in cells [21]. However, there is some controversy over whether NOS-like enzymes exist in higher plants [22]. There are homologues which have been found in algal species [23,24], but if higher plants have such an enzyme, its protein and gene(s) are being very elusive [25]. An enzyme which is known to make NO in plants is nitrate reductase, and this enzyme has been the focus of attention for several research groups [26,27,28,29].

Downstream of NO generation is also not without controversy. In animals, the classical pathway involves the generation of cGMP through the action of soluble guanylyl cyclase [30]. However, such pathways have recently been questioned in plants [31]. What is clear is that NO can lead to post-translational modification of proteins. Most commonly studied is the modification of thiol groups, so called S-nitrosation (otherwise called S-nitrosylation: for a recent overview of terminology to be used in plant NO research see [32]) [33,34]. Other modifications include tyrosine nitration [35]. Such modifications are often reversible and can be thought of as being akin to phosphorylation, where proteins can be toggled between functional states. 

It can be seen, therefore, that NO is a crucial signaling molecule in plants. It can be generated endogenously, be seen to interact with many signal transduction components, and has numerous physiological responses. In this Special Issue, authors were invited to contribute papers encompassing this field of biochemistry. 

## 2. Aspects of NO Metabolism

Life evolved in the presence of reactive compounds and many of these have been adopted as signaling molecules [36]. Looking at an ancient species, i.e., the lichen *Ramalina farinacea,* Expósito et al. [37] showed that NO production was likely to be dependent on NR. An inhibitor of NOS did not reduce NO levels in the lichen, whereas they reported the activity of NR to be 91 μU/mg protein, comparable with other systems.

The synthesis of NO in plants remains controversial [22], with the terminology to be used around NOS-like enzymes in plants recently being discussed [32]. In this Special Issue, Hancock and Neill [38] used a bioinformatic approach but failed to find evidence of an obvious NOS protein in plant databases. They also discussed how NO needs to interact with other reactive signaling molecules, a theme also picked up by Corpas et al. [39]. They, in a mini-review, discussed how NO is produced by peroxisome and that the NO produced interacts with glutathione and reactive oxygen species metabolism.

Two papers returned to the theme of NO production by discussing the enzyme nitrate reductase. Mohn et al. [40] reported on a comparative study between two NR isoforms, NIA1 and NIA2, and suggested that the different isoform have specialist functions. Tejada-Jimenez et al. [41] took a critical look at NR function and how interacting proteins may be involved. Lechón et al. [42] continued this theme by investigating the overproduction of NO in *cue1* mutants, and found that NO accumulation only occurs once seedlings are established. 

Downstream events in NO-mediated signaling are embraced in the remaining papers. Post-translational modification (PTM) of proteins via *S*-nitrosation was discussed by Corpas et al. [39] as part of their discussions on peroxisome, but PTMs were also discussed by Aranda-Caño et al. [43]. Here, the role of nitrate fatty acids (NO_2_-FAs) was discussed as signaling molecules and also how they may affect the modification of proteins, and hence function and activity. A second PTM, nitration, is a subject discussed by Takahashi and Morikawa [44]. In particular, they discussed the possible tyrosine nitration of PYR/PYL/RCAR receptors in leaves of *Arabidopsis thaliana*. Stimulated plant growth is the result of the signaling of PYR/PYL/RCAR receptors in *Arabidopsis thaliana,* and the authors studied how this may help to mediate the stimulation of plant growth.

The interaction of NO with glutathione and the formation of *S*-nitrosoglutathione is an immensely important aspect of NO biology [45]. Jahnová et al. [46] summarized the current thoughts on *S*-nitrosoglutathione reductase and how it has a crucial role in NO-based signaling.

## 3. Conclusions on *S*-Nitrosoglutathione Reductase (GSNOR) and How This Alters the Metabolism of *S*-Nitrosoglutathione and Hence *S*-Nitrosation of Proteins in Plant Cells

I hope that this Special Issue is a useful collection of papers which gives the reader an insight into the exciting area of NO biology in plants, and also hope that it inspires researchers to continue to work in this area, or indeed, to start investigations on plant NO metabolism. Such work would lead to the use of the manipulation of NO in plants as a way to enhance plant health and crop production, especially under stressful conditions [47].
